# Transient agonism of the sonic hedgehog pathway triggers a permanent transition of skin appendage fate in the chicken embryo

**DOI:** 10.1126/sciadv.adg9619

**Published:** 2023-05-17

**Authors:** Rory L. Cooper, Michel C. Milinkovitch

**Affiliations:** ^1^ Laboratory of Artificial and Natural Evolution (LANE), Department of Genetics and Evolution, University of Geneva, 1211 Geneva, Switzerland.; ^2^ SIB Swiss Institute of Bioinformatics, Geneva, Switzerland.

## Abstract

Vertebrate skin appendage early development is mediated by conserved molecular signaling composing a dynamical reaction-diffusion–like system. Variations to such systems contribute to the remarkable diversity of skin appendage forms within and among species. Here, we demonstrate that stage-specific transient agonism of sonic hedgehog (Shh) pathway signaling in chicken triggers a complete and permanent transition from reticulate scales to feathers on the ventral surfaces of the foot and digits. Resulting ectopic feathers are developmentally comparable to feathers adorning the body, with down-type feathers transitioning into regenerative, bilaterally symmetric contour feathers in adult chickens. Crucially, this spectacular transition of skin appendage fate (from nodular reticulate scales to bona fide adult feathers) does not require sustained treatment. Our RNA sequencing analyses confirm that smoothened agonist treatment specifically promotes the expression of key Shh pathway–associated genes. These results indicate that variations in Shh pathway signaling likely contribute to the natural diversity and regionalization of avian integumentary appendages.

## Teaser

Via transient embryonic manipulation of gene expression, scales are permanently replaced by feathers on the feet of chicken.

## INTRODUCTION

Vertebrate skin appendages are a diverse group of organs that adorn the integument, including scales, spines, feathers, hair, teeth, nails, and multiple exocrine glands (e.g., mammary and sweat glands) (*1*). Despite their natural diversity in form within and among species, vertebrate skin appendages share highly conserved early developmental processes. With the exception of mechanically patterned crocodilian face and jaw scales (*2*), skin appendages develop from anatomical placodes (*3*–*5*) whose spatial distribution and early differentiation are mediated by conserved molecular signaling (*1*, *5*–*9*) that form a system with reaction-diffusion–like dynamics (*10*–*13*).

One key player in vertebrate skin appendage development (*14*–*16*) is the sonic hedgehog (Shh) signaling pathway, known to mediate patterning, differentiation, and growth in many other developing systems, from the neural tube to the limb bud (*17*, *18*). Canonical Shh signaling begins with the SHH protein binding to its receptor, Patched1 (PTCH1). This reverses the repression of the transmembrane protein Smoothened (SMO) and activates the intracellular zinc finger protein GLI2, triggering subsequent downstream transcription and Shh pathway signaling (*19*, *20*). In the context of skin appendages, *Shh* expression is known to both specify flight feather positional information (*21*) and underpin feather form and diversification (*16*, *22*). Studies from mice have also demonstrated that perturbation of Shh signaling can result in morphogenetic defects during hair follicle development (*23*, *24*). Overall, examining the role of Shh signaling has markedly advanced our knowledge of vertebrate skin appendage development.

**Fig. 1.**
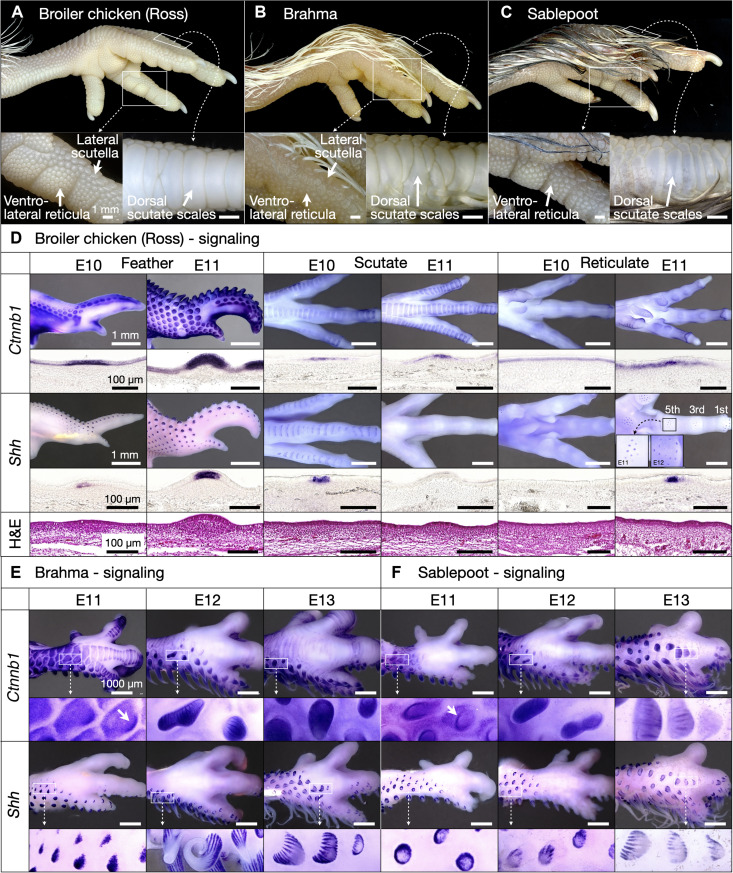
Developmental diversity and regionalization of chicken skin appendages. (**A**) Juvenile Broiler chickens display both feathers and three scale types, including small nodular reticula on the ventral surfaces of the foot and digits, scutella on the lateral digit surface, and large overlapping scutate scales on the dorsal foot surface and metatarsal shank. (**B** and **C**) Some special breed chickens, including the Brahma (B) and Sablepoot (C), exhibit feathers that emerge from scutate scales and scutella but not from reticula. (**D**) Normal development in Broiler chicken of wing feathers (left panels), scutate scales (central panels), and reticula (right panels) from placodes expressing *Ctnnb1* and *Shh*. On the wing, feather placodes visible at E10 continue propagating at E11. Gene expression is visible in early developing scutate scales at E10, continuing to undergo morphogenesis at E11. Local gene expression corresponding to individual reticulate scales first emerges at E11 on the first, third, and fifth toepads; insets show that *Shh* expression is transient in individual placodes, propagating from toepad centers to their periphery. Placodes develop at later stages on the second and fourth toepads. H&E staining (bottom row of panels) reveals localized thickening of the epidermis associated with placode development for all skin appendages. (**E** and **F**) Embryonic Brahma (E) and Sablepoot (F) chickens exhibit early feather buds (visible as zones of increased *Ctnnb1* and *Shh* staining; white arrows) emerging upon scutate scales at E11 and subsequently undergoing outgrowth. At E12 and E13, these foot feathers display characteristic striated expression of *Shh* associated with feather branching morphogenesis (*22*).

The chicken embryo has served as an important model for understanding skin appendage development (*25*). Feathers are the most widely studied chicken skin appendage type (*26*, *27*), and recent findings have demonstrated that their spatial patterning across the skin field depends upon the integration of mechanical and molecular interactions (*13*, *28*). Additional research in chicken has focused on the development of (i) the large, overlapping scutate scales adorning the shanks and dorsal foot surfaces (*29*, *30*), and (ii) the nodular reticulate scales (reticula) found upon the ventral surfaces of the foot and digits (*30*–*32*). This dorsoventral distinction in scale form is reportedly mediated by expression of both the engrailed homeobox 1 (*En-1*) and LIM homeobox transcription factor 1 (*Lmx1*) (*33*). Furthermore, some avian species including special breed chickens, such as the Brahma and Sablepoot varieties (Fig. 1), exhibit an anomalous polygenic trait called ptilopody, characterized by feathers adorning the shanks and dorsal foot surfaces (*34*). Recent genomic analyses revealed that perturbed expression of paired like homeodomain 1 (*Pitx1*) and ectopic expression of T-box transcription factor 5 (*Tbx5*) are associated with abnormal dorsal foot feather development in both pigeons and chickens, suggesting that ptilopody evolved independently in these species (*35*, *36*). Although there are variations in skin appendage regionalization both within and among different species, all avian skin appendage types are considered placode-derived and are patterned according to reaction-diffusion–like dynamics (*13*, *27*, *31*).

Previous research has demonstrated that various molecular switches can alter skin appendage fate in the chicken embryo, triggering the development of ectopic feathers upon both scutate and reticulate scales. Retinoic acid (RA) can induce such an effect, in a stage-dependent manner (*37*, *38*). However, the direct interactions of RA are diverse (*39*–*41*), meaning it is difficult to determine the pathways primarily responsible for the feather-on-scale development in this context. Similar phenotypes were reported from studies using replication-competent avian sarcoma (RCAS) viral vectors to induce misexpression of *Ctnnb1* (i.e., the gene coding for the β-catenin protein) (*42*), Notch/Delta pathway activation (*43*), or suppression of the bone morphogenetic protein (BMP) pathway (*44*). More recently, RCAS experiments indicated that *Sox2*, *Zic1*, *Grem1*, *Spry2*, and *Sox18* can also induce feather bud growth upon scutate scales (*45*). However, among these genes, only *Sox18* produced “true” feathers with associated follicles, branching, and keratinization (*45*). In none of these studies (*37*, *42*–*45*) has the specific role of Shh pathway signaling in foot scale versus feather development been investigated. Furthermore, as the postembryonic development of ectopic feathers resulting from such experiments has not, to our knowledge, previously been examined, it remains unclear whether they constitute transient abnormalities or permanent phenotypic transitions.

Here, we use a single intravenous injections of smoothened agonist (SAG) at embryonic day 11 (E11) to specifically promote Shh pathway signaling in the chicken embryo (*46*). We show that this treatment is sufficient to trigger the emergence of abundant ectopic feathers in areas that would normally form reticula on the footpad and ventral digit surfaces. Contrary to ectopic feather emergence from treatment with RA (*37*), the ectopic feathers we observe after manipulation of the Shh pathway do not develop upon individual reticula but instead emerge directly from the skin in loosely organized clusters associated with each toepad, suggesting a complete reticula-to-feathers transition. Incidentally, this treatment also induces ectopic feather emergence directly upon scutate scale ridges. Hematoxylin and eosin (H&E) staining and whole-mount in situ hybridization (WMISH) show that the ectopic feathers that we observe upon manipulation of the Shh pathway are developmentally comparable to normal feathers adorning the body.

Next, we use RNA sequencing (RNA-seq) of a time series of SAG-treated versus control embryos to examine the molecular mechanisms underlying the observed scale-to-feather conversion. These analyses confirm the specificity of the agonism exerted by SAG on the Shh pathway signaling.

Examination of their postembryonic development reveals that these ectopic feather follicles initially produce down-type feathers and subsequently mature to give rise to bilaterally symmetric contour feathers in adult chickens. These results demonstrate that Shh pathway agonism triggers the alteration of skin appendage placode fate, giving rise to a complete and permanent transition of skin appendage type, i.e., from reticulate scale to feather. As SHH is not only an important developmental morphogen, but also a growth and differentiation inducer in wide ranging contexts (*14*, *17*, *21*), we suggest that variations to its signaling contribute to the natural diversity and regionalization of avian integumentary appendages.

## RESULTS

### The developmental diversity of skin appendages in chickens

Feathers adorn the bodies of chickens, with the metatarsal shank and feet remaining uncovered. These latter regions instead display diverse, precisely patterned scale types (Fig. 1A). The ventral side of the footpad and digits are covered with small, nodular reticula, and the lateral digit surface is covered with slightly larger scutella, whereas the metatarsal shank and dorsal foot surface are covered with large overlapping scutate scales. However, some naturally occurring special breed chickens, including the Brahma and Sablepoot varieties, display feathers that emerge upon scutate scales of the dorsal foot surface and metatarsal shank (Fig. 1, B and C) (*34*–*36*). We first sought to examine the development of these distinct skin appendage types in different chicken breeds.

We use WMISH for classic markers of epidermal appendage initiation and morphogenesis to compare the normal development of feathers, scutate scales, and reticulate scales in the broiler (Ross) chicken (Fig. 1D). *Ctnnb1* and *Shh* staining reveals early-stage feather buds propagating across the wing at E10 (Fig. 1D, left panels) (*1*). By E11, feather buds cover the entire wing, undergoing varying stages of morphogenesis. Expression of both *Ctnnb1* and *Shh* associated with scutate scale patterning (Fig. 1D, central panels) is observed at E10; however, only expression of *Ctnnb1* persists to E11, as *Shh* is specifically expressed during the earliest development of these units. Transient local gene signaling associated with the development of individual reticula is observed at E11, with individual placodes expressing *Shh* at this stage (Fig. 1D, right panels) but not at E12, when additional peripheral *Shh*-positive placodes appear (inset of Fig. 1D, right panels). Hence, *Shh* expression in both scutate and reticulate scale primordia is substantially more transient than in feather primordia. Cryosections of WMISH samples reveal classical epidermal expression patterns of *Ctnnb1* and *Shh* (*5*), including the spatial restriction of *Shh* expression to the placode centers. H&E staining of paraffin sections (Fig. 1D, lower panels) reveals a local thickening of the epidermis associated with the developmental onset of these appendage types: the anatomical placode (*3*–*5*).

Next, we undertake WMISH for these same markers using embryos of Brahma and Sablepoot special bread chickens, which exhibitectopic feathers on their metatarsal shank and dorsal foot surfaces. In Brahma chicken embryos (Fig. 1E, left panels), *Ctnnb1* expression reveals a wave of scutate scales propagating across the shank and digits at E11. Note that scutate scale development at E11 is substantially more advanced in the Brahma chicken than in the broiler chicken (Fig. 1D). In addition, at E11, early feather buds are present on the feet of Brahma chicken embryos, with units appearing on the anterior edges of developing scutate scales (Fig. 1E, left panels, white arrows). By E12 and E13, feather buds in Brahma embryos propagate across the foot and become elongated. Although *Shh* expression is also observed in these ectopic feather buds of Brahma chicken embryos from E11 to E13, it does not stain scutate scales, in concurrence with its expression being restricted to earlier developmental stages of these units (Fig. 1D, central panels). By E12, the striated pattern of *Shh* expression associated with feather branching morphogenesis can be seen on developing ectopic feathers (Fig. 1E, central and right panels) (*22*). Although scutate scale emergence begins earlier in the Sablepoot chicken, meaning that expression of *Ctnnb1* has largely diminished by E11 (Fig. 1F), results are broadly comparable to those observed in the Brahma chicken, with feather buds developing upon scutate scales at E11 and a striated pattern of *Shh* expression appearing at E12.

Feather buds on the feet of either special breed chicken exhibit marked variation in their shapes, sizes, and spacing, often fusing together to form enlarged units (Fig. 1, E and F). Overall, expression patterns of *Ctnnb1* and *Shh* associated with development of foot feathers appear to be comparable between these two special breeds, as units develop in a wave across the metatarsal shank and dorsal digits, following otherwise normal development of the scutate scales (Fig. 1, E and F). On the other hand, the genomic mutations causing the emergence of ectopic feathers in Brahma and Sablepoot chicken do not seem to affect the spatial patterning nor the morphogenesis of reticula as the latter appear unmodified in these special breeds (i.e., in comparison to those of the broiler chicken; Fig. 1, A to C).

### SAG treatment triggers a reorganization of the avian integument, altering skin appendage fate and regionalization

**Fig. 2.**
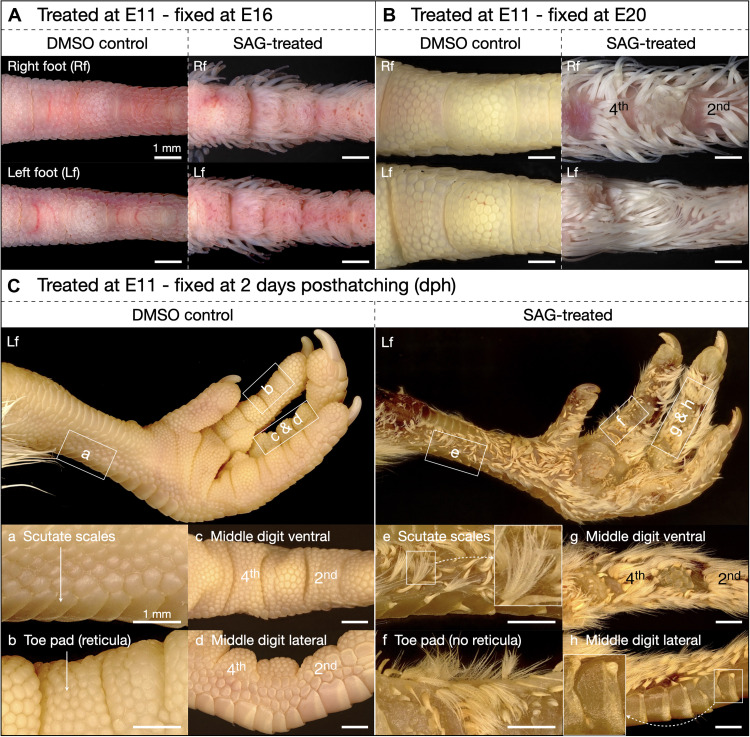
Agonism of Shh pathway signaling alters skin appendage fate and regionalization. Samples at E11 were treated with a single intravenous injection of either DMSO (controls) or 200 μg of SAG, before subsequent fixation and imaging at different stages. (**A**) Control samples (left panels) fixed 5 days after treatment (E16) reveal normal patterning of reticula on the ventral digit surface, whereas SAG treatment (right panels) triggers abundant ectopic feather bud development on both ventral and lateral digit surfaces, in regions that would normally form reticula and scutella. (**B**) Control samples (left panels) fixed 9 days after injection (E20) also exhibit normal scale patterning, whereas SAG-treated samples (right panels) display substantial coverage of keratinized ectopic feathers and absence of reticula. (**C**) Control samples (left panels) fixed 12 days after treatment [2 days posthatching (dph)] exhibit normal scale patterning of the foot and metatarsal shank, while SAG-treated samples (right panels) reveal extensive coverage of ectopic down-type feathers, including feathers on the ventral and lateral digit surfaces in place of reticula (f and g) and scutella (e and h), respectively. Feathers also emerge from the leading edges of scutate scales (e and h). Images of all replicates (Table 1) are shown in figs. S1 to S3.

We next sought to experimentally alter *Shh* signaling during reticula development, by treating chicken embryos with a single intravenous injection of SAG (see Materials and Methods for further details and Table 1 for a summary of replicates). Each injection was undertaken at E11, to coincide with the propagation of reticulate scale placodes on the ventral digits and footpad surface (Fig. 1). Control embryos injected with dimethyl sulfoxide (DMSO) at E11 and subsequently fixed at E16 exhibit normal development of nodular reticulate scales on the ventral footpad and digit surface (Fig. 2A, left panels). However, embryos injected with 200 μg of SAG show the emergence of abundant elongated ectopic feather buds from both the ventral and lateral digit surfaces (Fig. 2A, right panels). Contrary to previous reports of RA triggering development of ectopic feathers directly upon other avian scale types (*37*), here, we observe ectopic feather emergence and propagation directly from the epidermis, in areas where reticulate scales and scutella would normally arise. Note that reticula-to-feather transition upon SAG treatment is highly reproducible (fig. S1): Every surviving SAG-treated embryo (*n* = 12 of 20) shows substantial ectopic feather growth.

**Table 1.** Summary of replicates from SAG treatment experiments. dph, days posthatching.

Embryonic stage of treatment and fixation (days)	Figures in which data are presented	DMSO control survival (#)	SAG-treated survival (#)	Treatment effectiveness (%)
E11 to E16	Fig. 2 and fig. S1	8 of 9 (89%)	12 of 20 (60%)	100
E11 to E20	Fig. 2 and fig. S2	3 of 4 (75%)	9 of 20 (45%)	100
E11 to 2 dph	Fig. 2 and fig. S3	1 of 1 (100%)	3 of 6 (50%)	100
E11 to 110 dph	Fig. 6 and figs. S4 and S9 to S12	3 of 4 (75%)	4 of 12 (33%)	100

Embryos treated with SAG but fixed shortly before hatching at E20 reveal that ectopic feather buds undergo full keratinization (Fig. 2B). Once again, the result of the treatment is highly reproducible as every surviving embryo exhibits extensive ectopic feather growth (*n* = 9 of 20), although some variation in coverage is present (fig. S2), possibly because of subtle differences in embryonic stages at the moment of injection. The ectopic feathers that we observe in SAG-treated embryos persist in all surviving samples fixed at 2 days posthatching (dph) (*n* = 3 of 6) (Fig. 2C and fig. S3) or 3 dph (fig. S4), and, following their unfurling, they can be identified as down-type feathers (see inset in Fig. 2Ce), which lack a central ridge known as a rachis (*47*).

Furthermore, as well as observing ectopic feathers in regions that would normally form reticula and scutella, we also observe the development of ectopic feathers along the leading edges of scutate scales (e.g., inset of Fig. 2C, e and h). Therefore, in addition to altering skin appendage fate on the ventral and lateral digit surfaces, intravenous SAG treatment also triggers the emergence of ectopic feathers upon scutate scales undergoing morphogenesis.

**Fig. 3.**
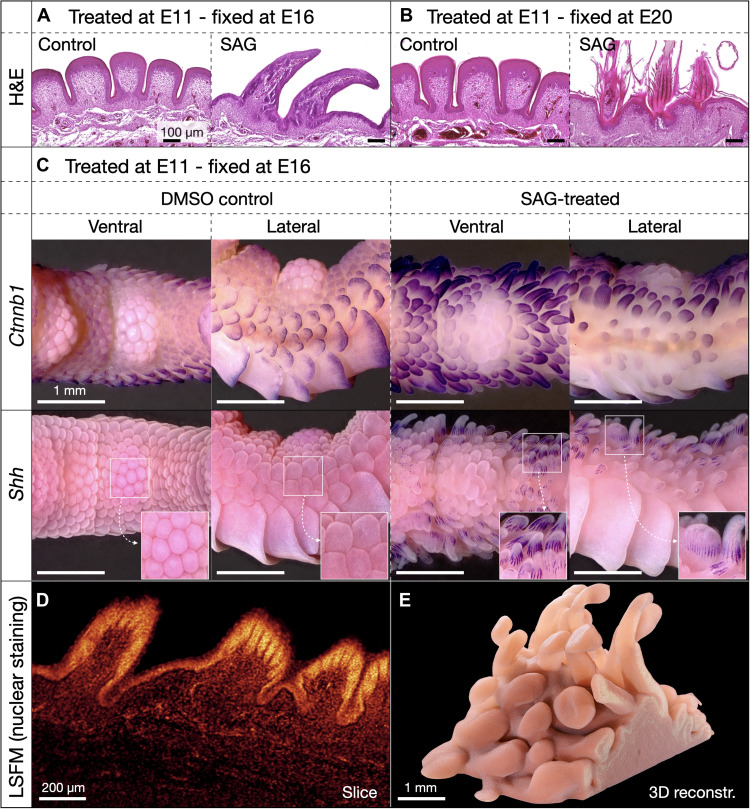
Development of SAG-induced ectopic feathers. (**A**) H&E-stained paraffin sections of samples fixed 5 days after treatment reveal normal nodular reticula in controls and typical feather-bud outgrowth in SAG-treated samples. (**B**) In control samples fixed at E20, regularly spaced, keratinized reticula are visible, whereas SAG-treated samples exhibit keratinized down-type feathers with multiple barbs. (**C**) WMISH at E16 reveals expression of *Ctnnb1* and absence of *Shh* in scutella undergoing morphogenesis in control samples (left panels), whereas SAG-treated samples (right panels) show developing ectopic feathers (on both ventral and lateral digit surfaces) with diffuse expression of *Ctnnb1* and characteristic striated expression of *Shh* associated with feather branching morphogenesis (*22*). (**D** and **E**) Light sheet fluorescence microscopy (LSFM) of a nuclear stained (TO-PRO-3 Iodide) sample fixed at E16 (i.e., 5 days after SAG treatment) shows both early feather branching morphogenesis (D, optical section) and the highly diverse shapes, sizes, and spacing of ectopic feather buds [3D reconstruction in (E); see also movie S1].

We next aimed to determine whether ectopic feathers resulting from treatment with SAG are developmentally comparable to normal feathers (Fig. 3). H&E staining of samples at E16 (Fig. 3A) reveals nodular reticulate scales of control samples and outgrowth of ectopic feather buds in SAG-treated samples. At E20 (Fig. 3B), H&E sections again reveal keratinized, nodular reticulate scales of control samples, whereas highly keratinous ectopic feathers consisting of multiple barbs have developed in SAG-treated samples. WMISH of samples fixed at E16 (Fig. 3C) shows expected gene expression patterns associated with normal reticula development in controls (left panels) and with normal feather development, including diffuse expression of *Ctnnb1*, and characteristic striated expression of *Shh*, in SAG-treated samples (right panels). Light sheet fluorescence microscopy (LSFM) of nuclear-stained ectopic feathers reveals a vast morphological diversity of feather buds resulting from SAG treatment (Fig. 3, D and E, and movie S1). All our results indicate that, although they exhibit substantial variation in their shapes, sizes, and spacing, ectopic feathers resulting from SAG treatment are developmentally comparable to normal feathers adorning the wings and bodies of chickens.

### SAG-induced ectopic feather emergence is associated with increased Shh pathway activity

**Fig. 4.**
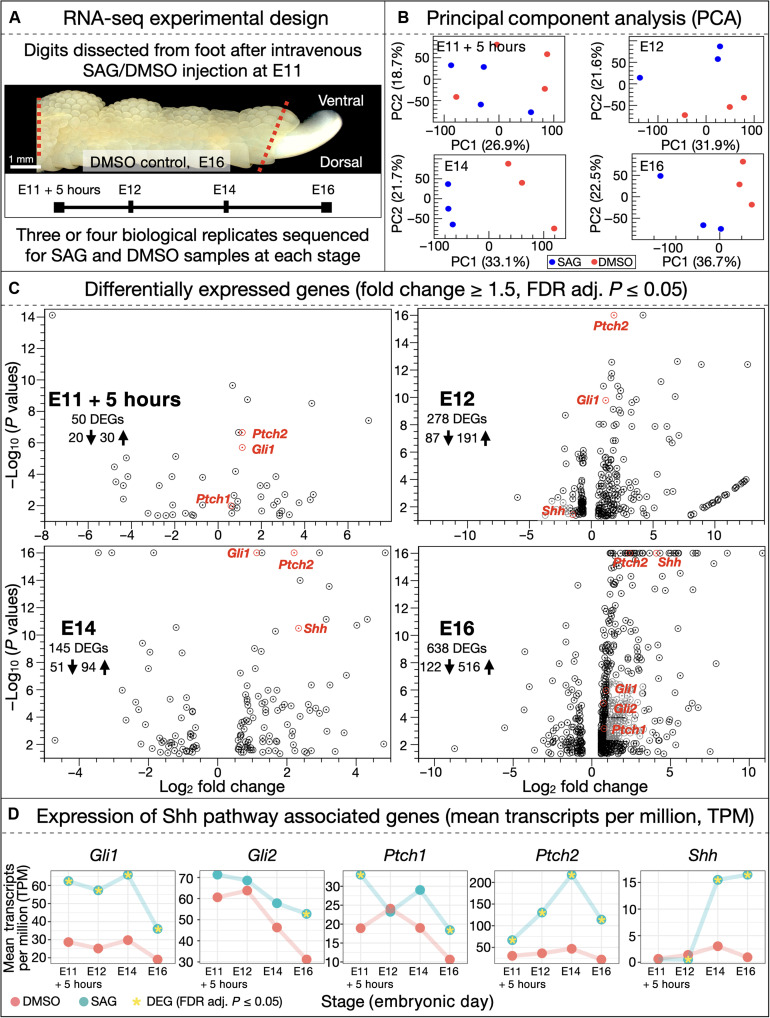
RNA-seq time course analysis of SAG-induced ectopic feather emergence. (**A**) Chicken embryos were injected with either SAG or DMSO at E11. Entire digits were subsequently dissected at multiple developmental stages (E11 + 5 hours, E12, E14, and E16) after treatment and processed for RNA-seq. (**B**) Principal component analyses (PCAs) are shown for each developmental stage at which RNA was extracted and sequenced, revealing separation of DMSO (red dots) versus SAG (blue dots) replicates. (**C**) Differentially expressed genes [DEGs; filtered by fold change ≥ 1.5 and false discovery rate (FDR) adjusted *P* ≤ 0.05] are shown in volcano plots for each stage. In SAG-treated samples, Shh pathway members *Gli1* and *Ptch2* are both up-regulated from E11 + 5 hours through to E16, expression of *Shh* is significantly up-regulated from E14 onward, and both *Gli2* and *Ptch1* are up-regulated at E16. (**D**) Individual expression values (in transcripts per million) are shown at each time point for Shh pathway–associated genes that are differentially expressed in at least one stage of our analysis (significant differential expression shown with an asterisk).

To examine the specific molecular mechanisms underlying the SAG-induced emergence of ectopic feathers, we used bulk RNA-seq analysis. Chicken embryos at E11 were injected with either DMSO (as a control) or SAG. RNA was subsequently extracted and sequenced from dissected digits at multiple times points after injection (Fig. 4A) (see Materials and Methods for further details and file S1 for analyzed RNA-seq datasets).

First, principal component analysis (PCA) reveals a clear separation in the PC1-PC2 plot between SAG-treated replicates (blue dots in Fig. 4B) and DMSO control replicates (red dots) from E12 onward, whereas the separation is not as clear yet at 5 hours after injection. Heatmaps also show the clustering of SAG-treated versus control samples at E12, E14, and E16 (fig. S5).

We next identify differentially expressed genes (DEGs) between SAG-treated and control samples (Fig. 4C). DEGs are filtered with a fold change ≥ 1.5 and a false discovery rate (FDR) adjusted *P* value ≤ 0.05. At E11 + 5 hours, 50 DEGs are identified (20 down-regulated and 30 up-regulated). Both the gene of the SHH receptor (PTCH2) and of the downstream target GLI1 are significantly up-regulated (*20*, *48*, *49*), indicative of increased Shh pathway activity, in the immediate hours following SAG injection. At E12, 278 DEGs are identified (87 down-regulated and 191 up-regulated). Note that the statistical significance of the *Ptch2* and *Gli1* up-regulation in SAG-treated digits is substantially larger than at the previous stage, indicating a sustained increase in Shh pathway activity. By E14, 145 DEGs are identified (51 down-regulated and 94 up-regulated). In addition to both *Ptch2* and *Gli1*, a strong up-regulation of *Shh* itself is also observed, suggesting that SAG treatment can trigger positive feedback of intrinsic Shh signaling. By E16, 638 DEGs are identified (122 down-regulated and 516 up-regulated), including up-regulation of Shh pathway–associated genes: *Gli1*, *Gli2*, *Ptch1*, *Ptch2*, and *Shh* itself (*20*, *48*, *49*). These results demonstrate that members of the Shh pathway are positively differentially expressed as a result of SAG treatment (Fig. 4C).

These results are confirmed in Fig. 4D by the observed temporal gene expression dynamics presented in mean transcripts per million; significant differential expression (FDR adjusted *P* ≤ 0.05) is noted with an asterisk. *Gli1* and *Ptch2* exhibit substantial up-regulation across all stages following SAG treatment. Expression of *Shh* itself increases rapidly after E12, and both *Gli2* and *Ptch1* are significantly up-regulated at E16. Note that changes in gene expression of other gene family members associated with skin appendage development (*1*) (fig. S6) are much less temporally consistent than changes in Shh pathway members. Some members of the Wnt (wingless/integrated), FGF (fibroblast growth factor), BMP, Dlx (distal-less homeobox), Sox (SRY-related HMG-box), and Hox (homeobox) families show some differential expression, although this is most often restricted to E16 (fig. S6), when the emergence of abundant ectopic feather buds is advanced (Fig. 2A). This also explains the substantial increase in expression, from E14 to E16, of feather keratin genes [especially keratin 1–like; *Fk1-l* (ENSGALG00010028542)] in SAG-treated digits (fig. S6), demonstrating that ectopic feathers are associated with true feather keratin types. Note that we also observe down-regulation of genes related to melanocyte production at E16 (fig. S6). Conversely to all these genes involved in the late development and differentiation of feathers, members of the Shh pathway appear to be causally involved in the observed scale-to-feather transition as they are more consistently up-regulated, including at early time points (Fig. 4D).

Although activation of either the RA pathway or the *Sox18* gene has been suggested to induce ectopic feathers in developing chicken embryos (*37*, *45*), we show here that SAG treatment does not yield any significant differential expression (FDR adjusted *P* ≤ 0.05), at any stage of our RNA-seq time course analysis, of either *Sox18* or the following key RA pathway–associated genes: RARs (RA receptors), RXRs (retinoid X receptors), *Raldh2* (retinaldehyde dehydrogenase 2), *Cyp26* (cytochrome P450 family 26), and CRABPs (cellular RA-binding proteins). Previous genomic studies have also shown that perturbed expression of *Pitx1* and ectopic expression of *Tbx5* are associated with the independent evolution of ptilopody in both pigeons and chickens (*35*, *36*). Again, we do not observe differential expression of these genes following SAG treatment, at any stage of our temporal RNA-seq analysis. Hence, our analyses indicate that SAG-induced ectopic feather emergence is specifically driven by up-regulation of the Shh pathway activity, i.e., a molecular mechanism that is distinct from those reported in previous studies (*35*–*37*, *45*).

We also used ingenuity pathway analysis (IPA; QIAGEN), using DEGs from each time point (filtered with FDR adjusted *P* ≤ 0.05), to examine whether SAG treatment yields the enrichment of specific pathways within the “organismal growth and development” IPA category (with human- and mouse-specific pathways removed; fig. S7). Besides confirming that Shh pathway signaling is increased (at E11 + 5 hours, E14, and E16), these analyses highlight the enrichment of other pathways of interest, most notably the planar cell polarity (PCP) and Wnt/βcat pathways, both enriched at E14 and E16. These results are coherent with previous studies indicating that feather bud branching morphogenesis requires PCP activity that, in turn, is promoted by Wnt/βcat pathway activity (*50*). The latter is also essential for diverse processes in feather bud development (*51*, *52*). The enrichment of BMP signaling at E16 and of epithelial-to-mesenchymal transition signaling across all time points (fig. S7) is expected, as their involvement is essential in skin appendage development (*7*, *53*). Overall, these enrichment analyses show that SAG treatment rapidly induces Shh pathway activity, whereas other gene families associated with feather bud development, including Wnt and BMP signaling, are enriched later, likely as a subsequent response to Shh pathway activity.

**Fig. 5.**
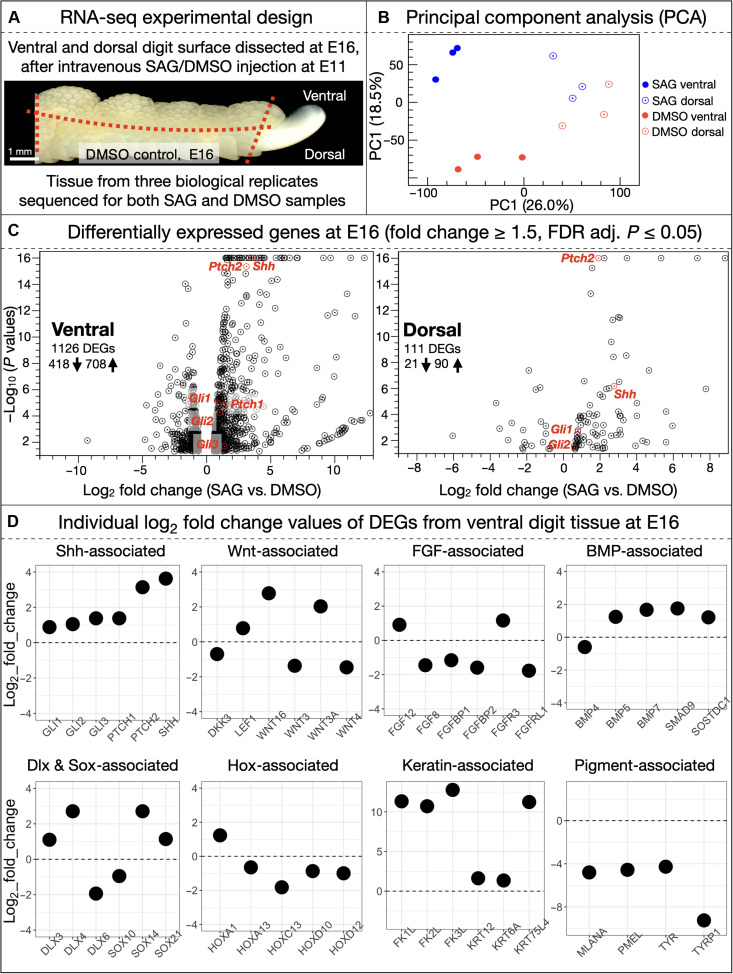
RNA-seq analysis of dorsal and ventral digit tissues after SAG treatment. (**A**) Chicken embryos were injected with DMSO (control) or SAG at E11. At E16, tissues from dorsal and ventral digit surfaces were dissected and processed for RNA-seq. (**B**) PCA reveals that dorsal versus ventral tissue samples separate along the PC1 axis (26% of total variance), whereas ventral replicates of SAG-treated versus DMSO controls separate along the PC2 axis (18.5% of variance). (**C**) Volcano plots reveal numerous DEGs in the SAG-treated ventral tissue, relative to the DMSO control (filtered by fold change ≥ 1.5 and FDR adjusted *P* ≤ 0.05). These DEGs include *Shh* itself as well as Shh pathway members *Gli1*, *Gli2*, *Gli3*, *Ptch1*, and *Ptch2*. Many fewer DEGs are observed in SAG-treated dorsal tissue; however, DEGs still include Shh pathway members *Gli1*, *Gli2*, *Ptch2*, and *Shh*. (**D**) Differential expression of selected genes associated with skin appendage development, in SAG-treated ventral digit tissues, is shown (filtered by fold change ≥ 1.5 and FDR adjusted *P* ≤ 0.05): Consistent up-regulation of Shh pathway–associated genes resulting from SAG treatment is accompanied by differential expression of individual Wnt-, FGF-, BMP-, Dlx-, Sox-, and Hox-associated genes, likely linked with ectopic feather bud emergence and morphogenesis. SAG treatment also causes the strong up-regulation of various keratin-associated genes (including those associated with feather keratin production) and down-regulation of various melanocyte-associated genes.

Next, as the SAG-induced scale-to-feather transition is most prominent on the ventral digit surfaces (Fig. 2A), we undertook comparative RNA-seq analyses of samples dissected at E16, from the ventral and dorsal digits in control and SAG-treated embryos (Fig. 5A). PCA reveals a clear separation of ventral versus dorsal tissues along PC1, and of ventral controls versus ventral SAG-treated samples along PC2 (Fig. 5B). A heatmap of all replicates also shows clear clustering of ventral samples, separated by treatment (fig. S8). Differential gene expression analysis (filtered by fold change ≥ 1.5 and FDR adjusted *P* ≤ 0.05) reveals 1126 DEGs (418 down-regulated and 708 up-regulated) in SAG-treated ventral tissue (Fig. 5C). Again, these include *Shh* and other members of the Shh pathway, such as *Gli1*, *Gli2*, *Gli3*, *Ptch1*, and *Ptch2*, confirming the significant up-regulation of Shh pathway signaling resulting from SAG treatment. Conversely, only 111 DEGS (21 down-regulated and 90 up-regulated) are identified in SAG-treated dorsal tissue, although *Gli1*, *Gli2*, *Ptch2*, and *Shh* are also up-regulated here; not an unexpected result given that SAG was injected intravenously and can therefore affect Shh pathway activity in all tissues. Whereas the five members of the Shh pathway listed above are up-regulated following SAG treatment (Fig. 5D, top left), we unsurprisingly observe various differential expressions of members from other gene families involved in skin appendage development (*1*), including Wnt, FGF, BMP, Dlx, Sox, and Hox (Fig. 5D, next five panels). Interestingly, we also observe in SAG-treated samples (Fig. 5D, two bottom right panels) strong up-regulation of various keratin-associated genes, including three feather keratin-like genes [*Fk1-l* (ENSGALG00010026851), *Fk2-l* (ENSGALG00010025898), and *Fk3-l* (ENSGALG00010028555)], and down-regulation of various genes associated with melanocyte production. These results from dissected ventral versus dorsal digit tissues are concurrent with those obtained from the RNA-seq time course analysis of full digits discussed previously (Fig. 4 and figs. S5 to S7): SAG treatment specifically up-regulates Shh pathway signaling.

### SAG-induced ectopic feathers constitute a permanent reorganization of the avian integument.

**Fig. 6.**
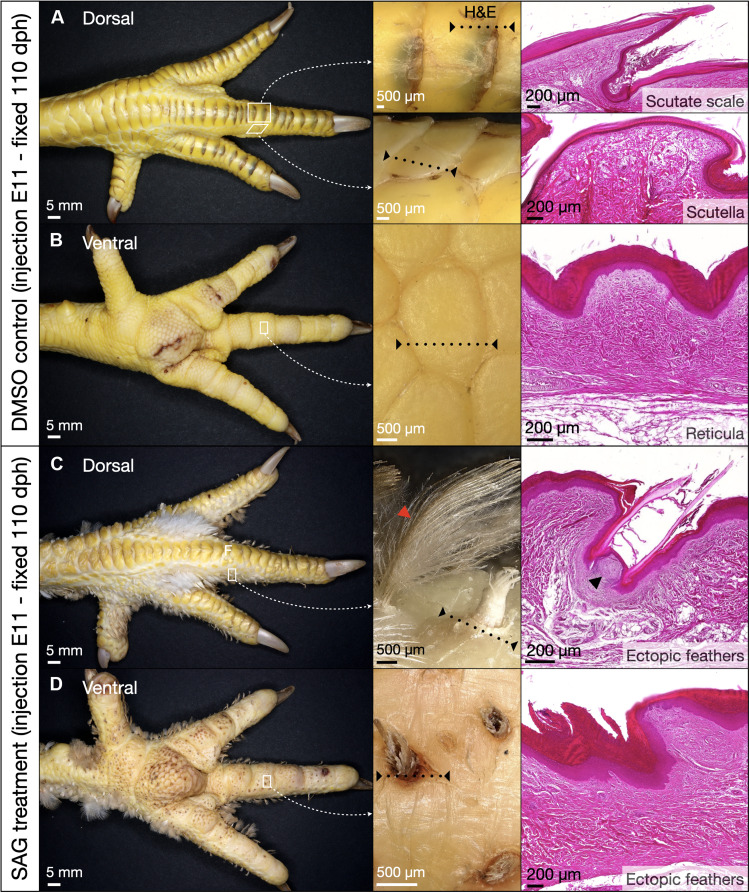
Sustained posthatching development of in ovo SAG-induced ectopic feathers. (**A** and **B**) Samples treated with a control injection of DMSO at E11 and fixed at 110 dph exhibit normal scale patterning (left and central panels) and normal histology (H&E-stained sections in right panels; dashed black lines in central panels show approximate locations), with large overlapping scutate scales on the dorsal foot surface and metatarsal shank (A), and smaller reticulate scales on the footpad and ventral digit surface (B). (**C**) Embryos treated with a single injection of SAG at E11 and fixed at 110 dph exhibit ectopic feathers (both down-type and bilaterally symmetric adult contour feathers, red arrowhead) emerging from scutate scale edges on the dorsal foot surface. (**D**) Although the most external parts of SAG-induced ectopic feathers on the ventral foot surface have been removed by abrasion during locomotion, feather follicles are clearly visible (central panel). In SAG-treated chickens (C) and (D), H&E sections reveal the keratinous calamus and associated feather follicle of ectopic feathers embedded within the skin, with the proliferative dermal papilla (C, right, black arrowhead) visible in some sections. All replicates are shown in figs. S9 to S12.

Last, we sought to examine the postembryonic development of SAG-induced ectopic feathers. Therefore, we tracked the progression of these feathers in hatched animals from 3 to 110 dph (Fig. 6 and figs. S9 to S12). At 3 dph, feather coverage on the ventral foot surface is extensive (figs. S4 and S9); however, by 8 dph, these units are much less clearly visible (fig. S10), and they appear to have virtually disappeared by 22 dph (fig. S11). We investigate the question of this apparent gradual disappearance of ectopic feathers in SAG-treated chickens by imaging the ventral foot tissues of these chickens after fixation at 110 dph (Fig. 6 and fig. S12). Control animals display normal patterning of avian scale types (Fig. 6, A and B). Conversely, SAG-treated animals reveal extensive ectopic feather coverage on the dorsal and lateral foot surfaces (Fig. 4, C and D). Upon closer inspection of the ventral foot surface, we note the total absence of reticulate scales and the presence of feather follicles (Fig. 6D), although feathers are either absent or damaged, because of abrasion during locomotion. All surviving SAG-treated chickens exhibit ectopic feathers and feather follicles (*n* = 4 of 12; fig. S12). We observe both down-type and contour feathers (*47*, *54*), with the latter having a central rachis supporting branching feather barbs (Fig. 6C, red arrowhead). H&E staining of ventral tissue containing ectopic feather follicles reveals the broken keratinous basal feather shaft known as the calamus, embedded within the epidermis of the foot surface (Fig. 6D). In some sections, a dermal papilla associated with cyclic growth is also apparent (Fig. 6C, black arrowhead) (*55*). These results demonstrate that SAG-induced ectopic feather follicles are comparable to normal follicles, which produce permanent, regenerative, bilaterally symmetric adult feathers (*47*).

## DISCUSSION

Previous research has demonstrated that the development of ectopic feathers upon foot scales in chicken embryos can be triggered by the perturbation of multiple signaling pathways (*37*, *42*–*45*). However, the feather phenotypes resulting from such experiments vary in terms of the presence or absence of a follicle, follicle invagination, branching geometry, and levels of keratinization, meaning that they likely cannot all be considered normal avian feathers (*45*). Furthermore, to our knowledge, the postembryonic development of these ectopic feather-like appendages has not previously been examined; hence, it remains unclear whether they constitute transient aberrations or permanent transitions (*45*).

The results that we report here demonstrate that SAG induces specific agonism of the Shh pathway and triggers the replacement of scales by bona fide feathers in regions of the avian foot that would normally form reticula and scutella (Fig. 2 and figs. S1 to S4). This result is distinct from reports of RA treatments inducing the development of ectopic feathers upon, and not in replacement of, reticulate scales (*37*). Furthermore, we show that SAG-induced ectopic feathers are developmentally comparable to normal feathers (Fig. 3) while exhibiting broad variation in their sizes and spacing, relative to the spatially ordered feathers on the body and wings (Fig. 1D).

Using RNA-seq, we demonstrate that SAG treatment results in rapid and persistent up-regulation of members of the Shh pathway (Figs. 4 and 5 and figs. S5 to S8), including a substantial positive feedback of intrinsic Shh signaling from E14 onward (Fig. 4D). These results indicate that the molecular mechanism underpinning SAG-induced scale-to-feather transition is distinct from previous findings, as we do not observe differential expression of genes associated with either the RA pathway or *Sox18* (*37*, *45*). Although we observe some transient up-regulation of other gene family members that are associated with skin appendage development (fig. S6), Shh pathway agonism appears to be the most consistent effect of SAG treatment across our time course analysis, therefore suggesting that Shh pathway activity primarily underpins ectopic feather emergence.

Last, we show that, like normal chicken feather follicles, the SAG-induced ectopic follicles initially produce down-type feathers, before subsequently producing bilaterally symmetric contour feathers in adult chickens (Fig. 6C) (*47*, *54*), including a dermal papilla associated with cycling growth and regeneration (*55*). Crucially, this transition of skin appendage fate (from reticula to bona fide feathers) does not require sustained drug treatment as a transient exposition to the *Shh*-agonist SAG, through a single intravenous injection at embryonic stage E11, is sufficient to produce the conversion that is then self-maintained. Therefore, the ectopic feathers resulting from this treatment are not merely transient anomalous embryonic structures but instead constitute permanent, regenerative skin appendages. Interestingly, these ectopic feathers appear to display greater morphological diversity in their sizes and spacing, when compared to precisely patterned feathers of the body or wing (Fig. 1D).

On the other hand, both RA and SAG treatments cause the emergence of ectopic feathers upon scutate scales ridges (Fig. 2C) (*37*), an effect reminiscent of ptilopody, i.e., a phenotype observed in certain special breed pigeons and chickens exhibiting feathers on their dorsal foot surfaces, emerging from the leading edges of scutate scales (Fig. 1, B, C, E, and F). Although ptilopody has previously been associated with modified expression of *Pitx1* and *Tbx5* (*35*, *36*), our RNA-seq analyses of chicken embryos treated with SAG do not reveal differential expression of these genes during scale-to-feather transition, suggesting that SAG-induced foot feathers development follows a distinct mechanism, primarily governed by increased Shh pathway activity (Figs. 4 and 5).

In conclusion, we have shown that transient agonism of the Shh signaling pathway, at the onset of reticulate scale embryonic development, is sufficient to trigger the emergence of abundant ectopic bona fide feathers upon regions of the avian foot surface that would otherwise exhibit distinctively patterned nodular scales. This reticula-to-feather conversion is stable as the ectopic feathers persist through postembryonic development, transitioning into regenerative, bilaterally symmetric adult feathers. Hence, our results demonstrate that the interaction network involved in the development of chicken skin appendages can be permanently shifted from one steady state (leading to the development of reticulate scales) to another steady state (leading to the development of feathers), opening the possibility to investigate how evolutionary changes in interaction networks allowed for marked transitions of forms among skin appendage types. Our study also indicates that natural variations in Shh signaling are likely such an evolutionary driver of scale and feather pattern diversity.

## MATERIALS AND METHODS

### Animal husbandry

Broiler chicken (Ross) eggs were obtained from the company La Prairie (1721 Cournillens, Switzerland). Eggs were incubated at 37.5°C with ~40% relative humidity. Sablepoot and Brahma chicken eggs were obtained from noncommercial specialist breeders. Maintenance of and experiments with all chicken embryos were approved by the Geneva Canton ethical regulation authority (authorization GE10619B) and performed according to Swiss law. These guidelines meet the international standards. Imaging of chickens was undertaken using a Keyence VHX 7000 digital microscope (Figs. 1 to 3 and figs. S1 to S4) or a mounted Nikon D800 camera (Fig. 4 and figs. S9 to S12).

### WMISH and cryosectioning

Embryos were fixed overnight in 4% paraformaldehyde (PFA) at 4°C and subsequently dehydrated into methanol (MtOH). WMISH was undertaken as previously described (*56*). Samples were postfixed in 4% PFA and imaged using a Keyence VHX 7000 digital microscope, before optimal cutting temperature compound embedding and cryostat sectioning (CM1850, Leica Microsystems, Wetzlar, Germany). Imaging of sections was undertaken with an automated slide scanner (Pannoramic Midi, 3DHISTECH, Budapest, Hungary).

### H&E staining of paraffin sections

Fixed samples were dissected and processed for H&E staining as previously described (*56*). Paraffin sections (10 μm) were acquired with a microtome (Leica RM2255). Stained sections were mounted using ultra kit mounting medium, and imaging was undertaken using an automated slide scanner (3DHISTECH).

### In vivo treatment with SAG

Eggs at E11 were cleaned with 70% ethanol and candled to identify suitable veins for injection. The largest vein observed from candling of the egg was targeted for injection. The eggshell covering the selected vein was removed using a detailing saw (Micromot 50/E, Proxxon, Wecker, Luxemburg) while keeping the underlying egg membrane intact. After removing the eggshell, mineral oil was applied to increase the transparency of the exposed membrane, and a candling torch was used to illuminate the window. SAG (Selleckchem, S7779) was dissolved in warm DMSO to make a stock solution of 50 μg/μl. The total weight of chicken eggs (and therefore embryos) varies depending on the age of laying hens. Therefore, SAG dosage was adjusted proportionally to account for differences in egg weight. For results shown in Figs. 2, 3, and 6, eggs weighted approximately 70 g. These eggs were treated with 4 μl of the SAG stock solution added to 26 μl of DMSO, resulting in a total of 200 μg of SAG in each 30-μl injection. For RNA-seq results shown in Figs. 4 and 5, egg weight was approximately 60 g. These eggs were treated with 3.4 μl of the SAG stock solution added to 26.6 μl of DMSO, resulting in a total of 170 μg of SAG in each 30-μl injection. The SAG solution was injected directly into the vein using a Hamilton syringe and a micromanipulator (MM33 right, Marzhauser, Wetzlar, Germany). Each embryo was treated with a single 30-μl injection. To enable clear visualization of the solution during injection, Patent Blue V (Sigma-Aldrich) was also added to the solution. Control embryos were injected with DMSO and patent blue only. Clear adhesive tape was applied to the window to prevent infection after injection, and eggs were incubated until the desired developmental stage. Embryos were subsequently fixed overnight in 4% PFA. The treatment was 100% effective in surviving embryos. All replicates are shown in figs. S1 to S4 and S9 to S12. SAG injections before E11 resulted in 100% embryonic mortality, whereas injections after E11 did not result in a consistent scale-to-feather conversion. Note that doses of 100 μg of SAG did not result in a scale-to-feather conversion, whereas doses of 300 μg of SAG resulted in total embryo mortality (see note added in proof).

### Light sheet fluorescence microscopy

Samples in methanol (MeOH) were rehydrated and permeabilized in phosphate-buffered saline with gelatine, sodium azide, saponin, and Triton X-100. Samples were then incubated in TO-PRO-3 Iodide (2:1000; Thermo Fisher Scientific, Waltham, MA, USA) for 4 hours, to stain cell nuclei. Following staining, samples were cleared according to the iDISCO protocol (*57*). Imaging was undertaken using a light sheet microscope (Ultramicroscope 2 Blaze, Miltenyi Biotec). LSFM data were processed using Fiji (*58*), before visualization with Imaris (Oxford Instruments, UK) (Fig. 3D). Furthermore, three-dimensional (3D) reconstructions of LSFM data were visualized using Houdini 3D procedural software (SideFX, Toronto, Ontario, Canada) to show the surface geometry of the samples. First, image stacks were reconstructed into a signed distance field volume that was then converted into a polygonal mesh to which global lighting and shader effects were applied to modify parameters including color, reflection, transparency, and subsurface scattering. Tissue layers from resliced image stacks were projected onto the exposed model sides using a nonrigid alignment. The result was rendered both as still images (Fig. 3E) and as a short animation (movie S1).

### RNA-seq experiments and analysis

Bulk RNA-seq was used to determine the molecular mechanisms underpinning SAG-induced scale-to-feather conservation. Entire digits (excluding the claw) were dissected from both the left and right foot of embryonic chickens treated at E11 with either DMSO (control) or SAG. Tissues from at least three biological replicates per treatment were collected at E11 + 5 hours, E12, E14, and E16 (data shown in Fig. 4 and figs. S5 to S7). Tissues were also collected from dissected dorsal and ventral digit regions at E16 (data shown in Fig. 5 and fig. S8). Dissected tissue was immediately placed in TRIzol (Sigma-Aldrich) and stored at −80°C. RNA was extracted using the Direct-zol RNA Miniprep Kit (Zymo Research). RNA-seq [Illumina TruSeq stranded mRNA - NovaSeq 6000 100 PE (paired end) with 40 M reads] was undertaken by Macrogen Europe (Amsterdam, The Netherlands). Both alignment of reads (to *Gallus gallus* genome bGalGal1.mat.broiler.GRCg7b) and analyses were undertaken in CLC Genomics Workbench (QIAGEN). Pathway enrichment was undertaken with IPA (QIAGEN), for DEGs at each stage of the time course analysis. Plots shown in figs. S6 and S7 were made using ggplot2 in R (*59*). All RNA-seq datasets analyzed in this study are provided in file S1, and raw RNA-seq data are provided at https://doi.org/10.5061/dryad.jwstqjqff.

*Note added in proof*: After the manuscript was accepted for publication, the authors requested that readers be made aware of an additional paper they authored, which includes further details regarding the protocol for egg injection: R. L. Cooper, G. Santos Durán and M. C. Milinkovitch, Protocol for the rapid intravenous in-ovo injection of developing amniote embryos, STAR Protocols, In Press (2023).

## Notes

View/request a protocol for this paper from *Bio-protocol*.
