# Supplementary material for: Transient agonism of the sonic hedgehog pathway triggers a permanent transition of skin appendage fate in the chicken embryo
Source: Sci Adv. 2023 May 17;9(20):eadg9619. doi: 10.1126/sciadv.adg9619 (PMC10191425; doi:10.1126/sciadv.adg9619)
Supplement: Supplementary file 1 — Figs. S1 to S12 Legend for movie S1 Legend for file S1 [file sciadv.adg9619_sm.pdf]

Science Advances
AAAS

Supplementary Materials for

Transient agonism of the sonic hedgehog pathway triggers a permanent transition of skin appendage fate in the chicken embryo

Rory L. Cooper and Michel C. Milinkovitch

Corresponding author: Michel C. Milinkovitch, michel.milinkovitch@unige.ch

Sci. Adv. **9**, eadg9619 (2023)
DOI: 10.1126/sciadv.adg9619

The PDF file includes:

Figs. S1 to S12
Legend for movie S1
Legend for file S1

Other Supplementary Material for this manuscript includes the following:

Movie S1
File S1

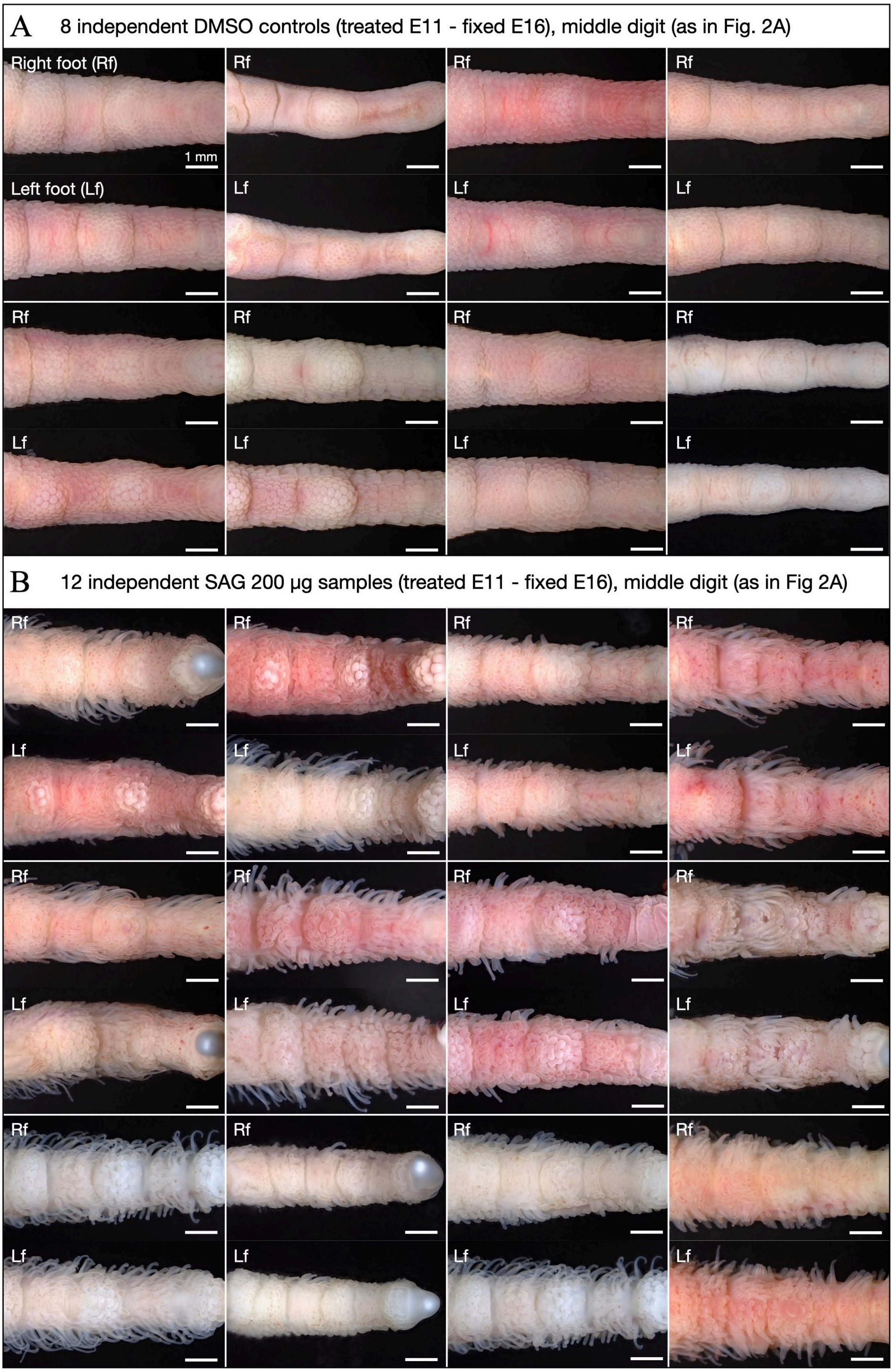

Supplemental figure S1. Replicates of control and SAG treatments (one single injection at E11) followed by fixation and imaging at E16. (**A**) DMSO-treated control samples show normal reticulate scale development. (**B**) SAG-treated samples exhibit ectopic feather development on the ventral and lateral digit surfaces.

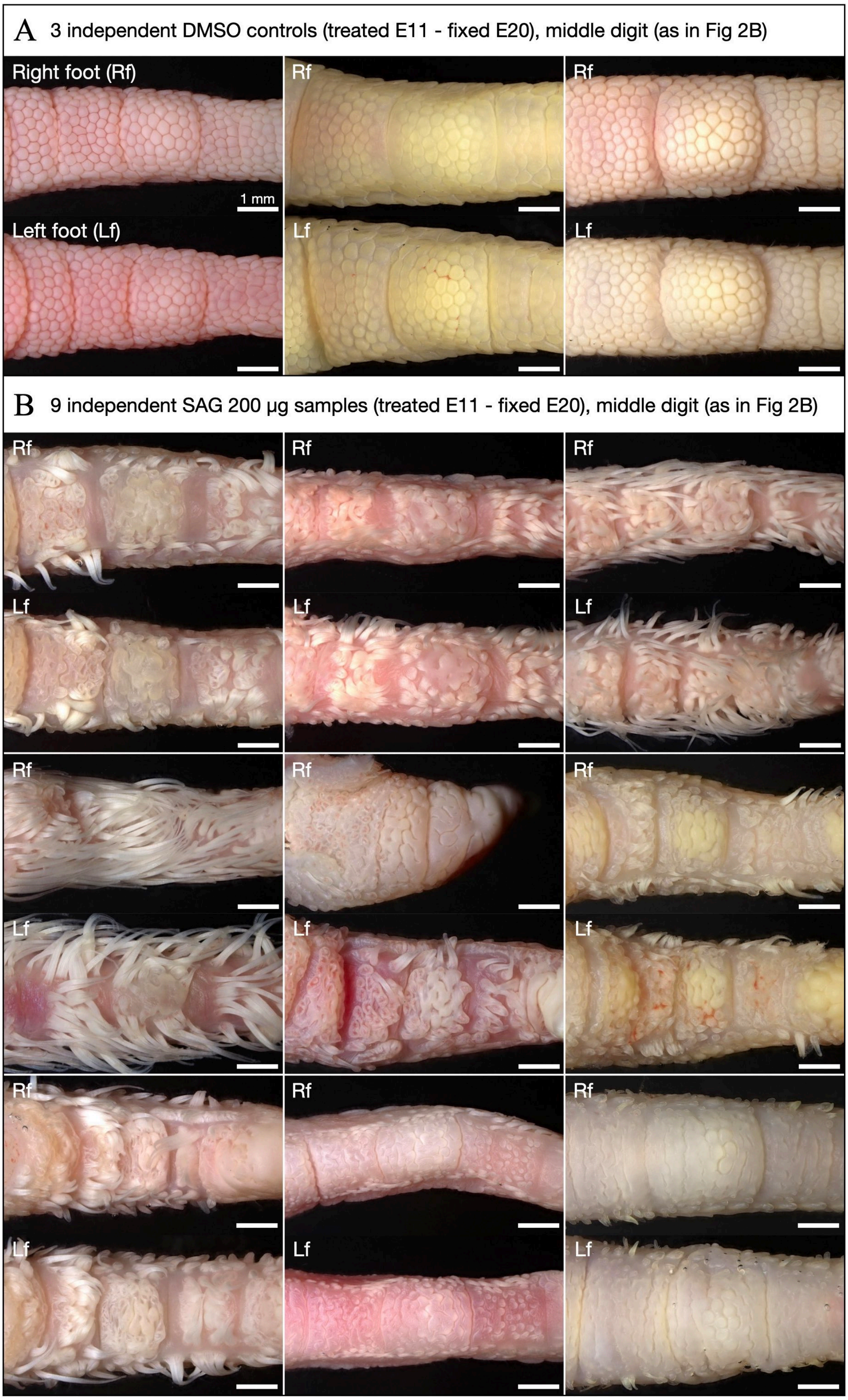

Supplemental figure S2. Replicates of control and SAG treatments (one single injection at E11) followed by fixation and imaging at E20. (**A**) Control samples show normal reticulate scale development. (**B**) SAG-treated samples exhibit the development of keratinised ectopic feathers on the ventral and lateral digit surfaces. The nine samples illustrate the variation in feather bud coverage, possibly because of subtle differences in embryonic stages at which the SAG treatment was applied.

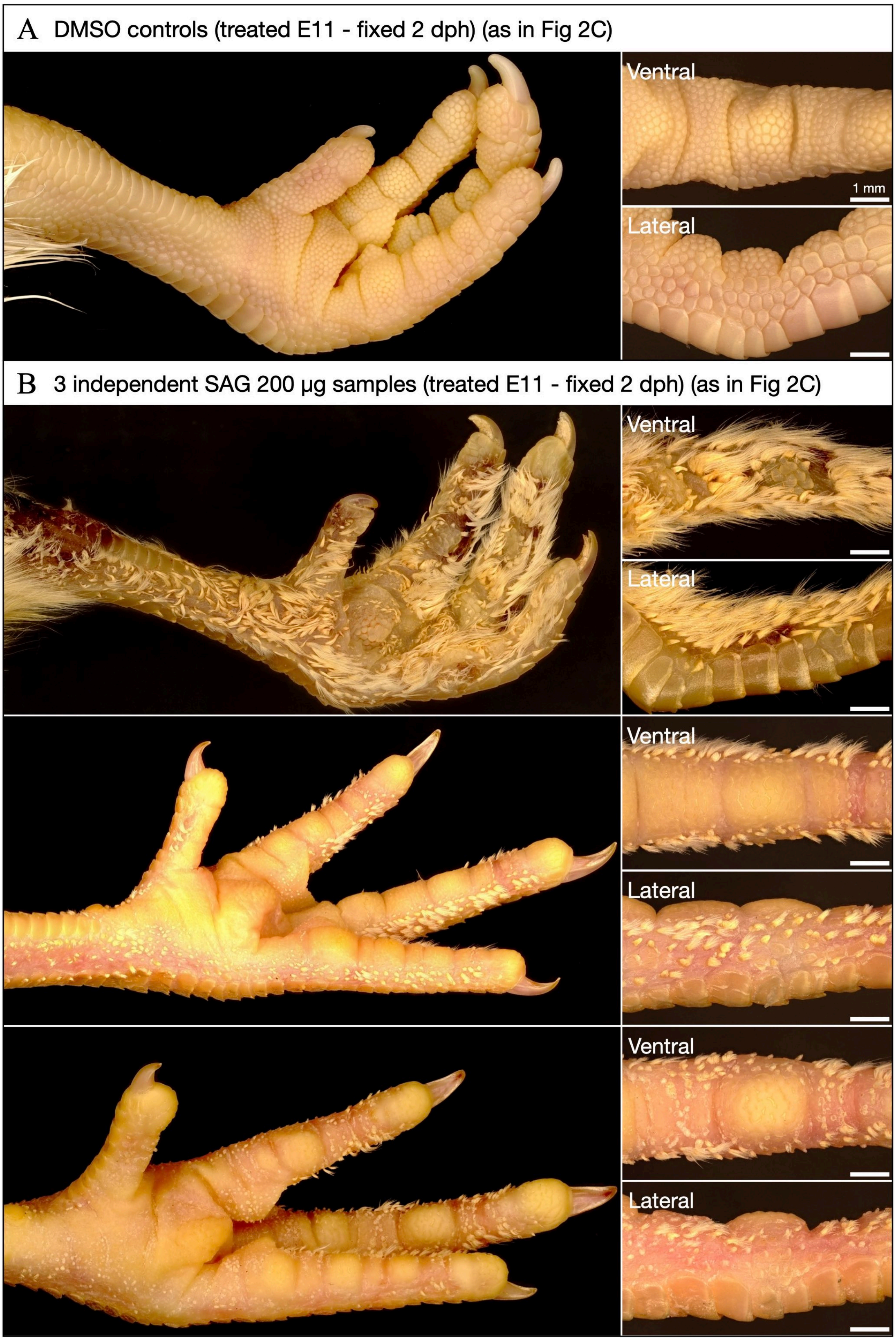

Supplemental figure S3. Replicates of control and SAG treatments (one single injection at E11) followed by fixation and imaging at 2 days post hatching (dph). (**A**) The DMSO-treated control sample shows normal scale development associated with the foot and metatarsal shank. (**B**) SAG-treated samples exhibit the development of keratinised ectopic feathers on the ventral and lateral digit surfaces, as well as ectopic feathers emerging from scutate scale edges. The three samples illustrate the variation in feather coverage, possibly because of subtle differences in embryonic stages at which the SAG treatment was applied.

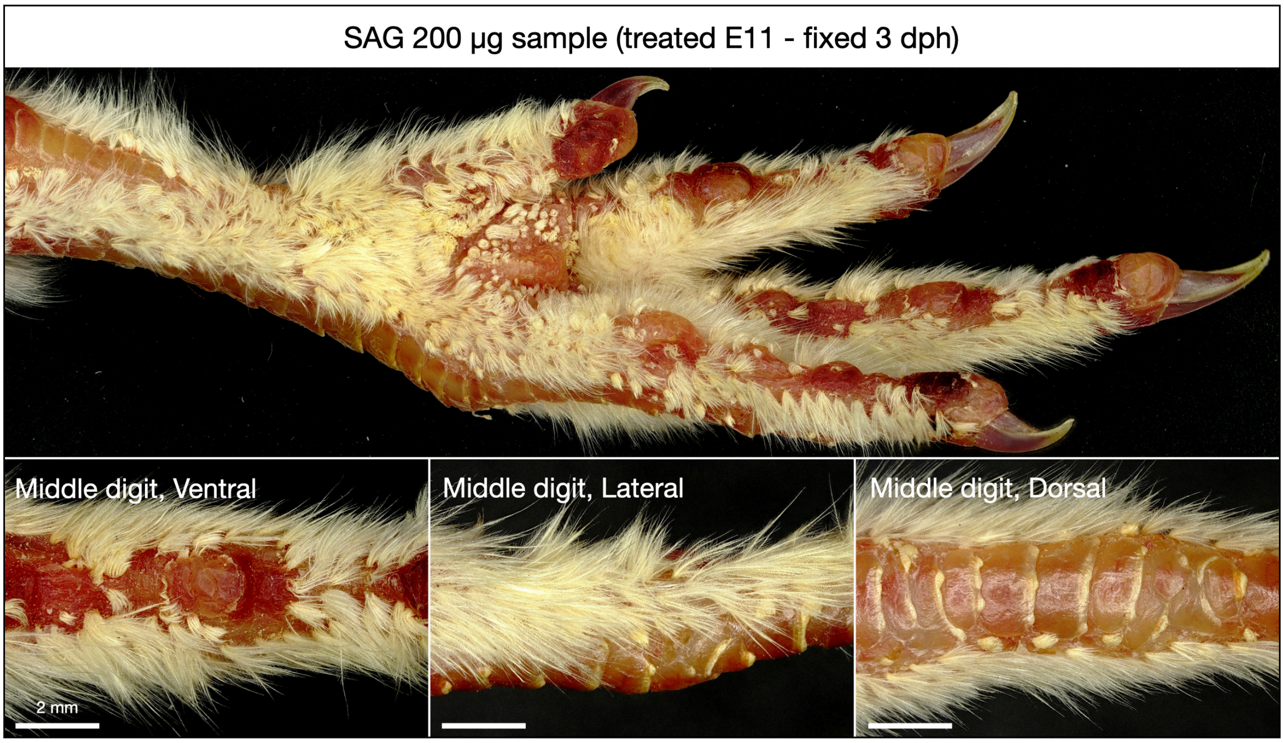

Supplemental figure S4. Replicate of SAG treatment (one single injection at E11) followed by fixation and imaging at 3 days post-hatching (dph). Extensive ectopic down-type feathers cover the ventral and lateral footpad surfaces. Others ectopic feathers emerge from the edge of scutate scales on the dorsal foot surface.

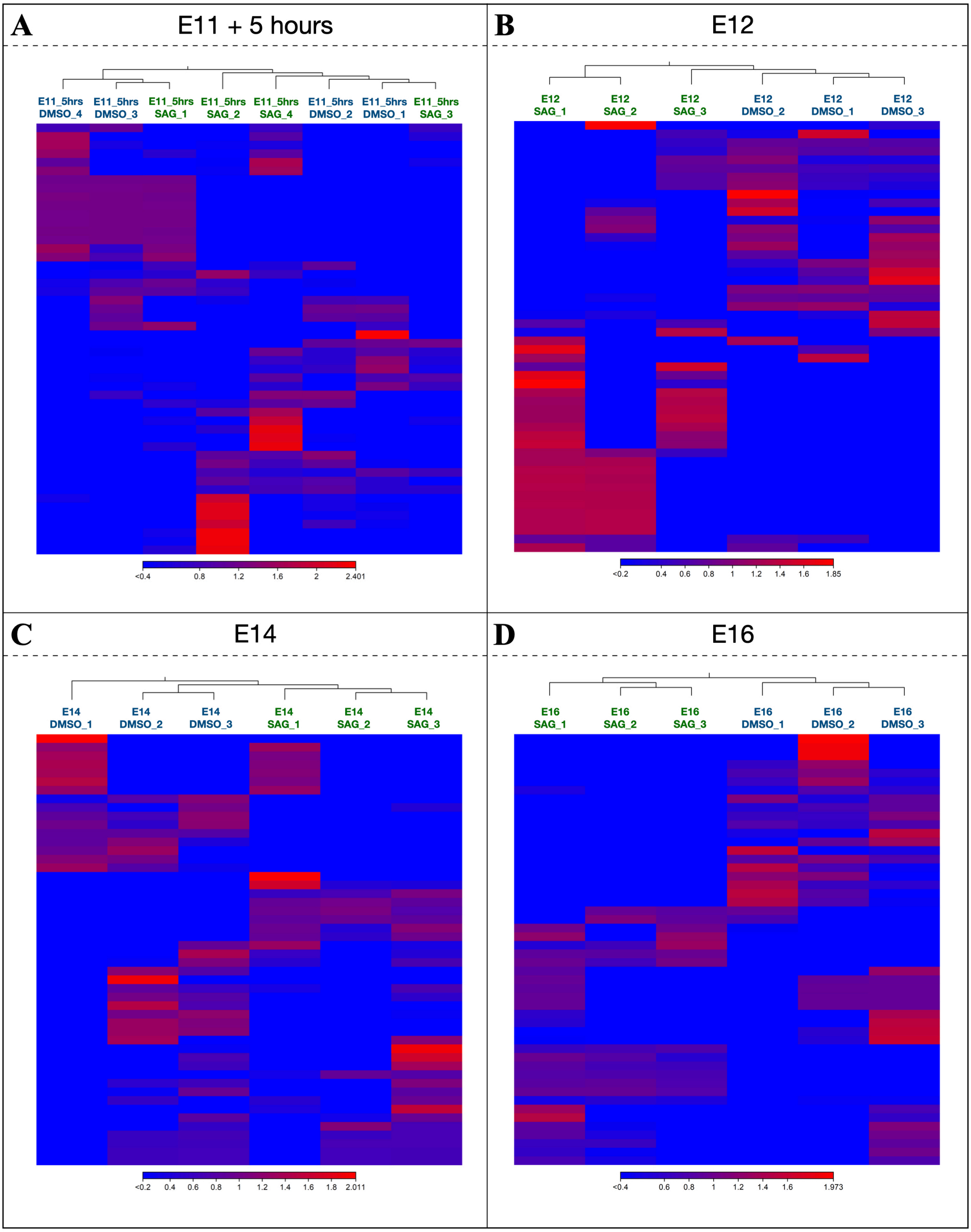

Supplemental figure S5: Heatmaps of replicates from RNA sequencing time course analysis, separated by stage. Heatmaps are generated with Euclidean distance, clustered by average linkage and filtered by 50 fixed features with a minimum of 10 counts. Samples do not cluster clearly at E11 + 5 hours, however clustering of both SAG treated and DMSO control digits is clear by E12 onwards. Differential gene expression analysis of these samples is shown in Fig. 4.

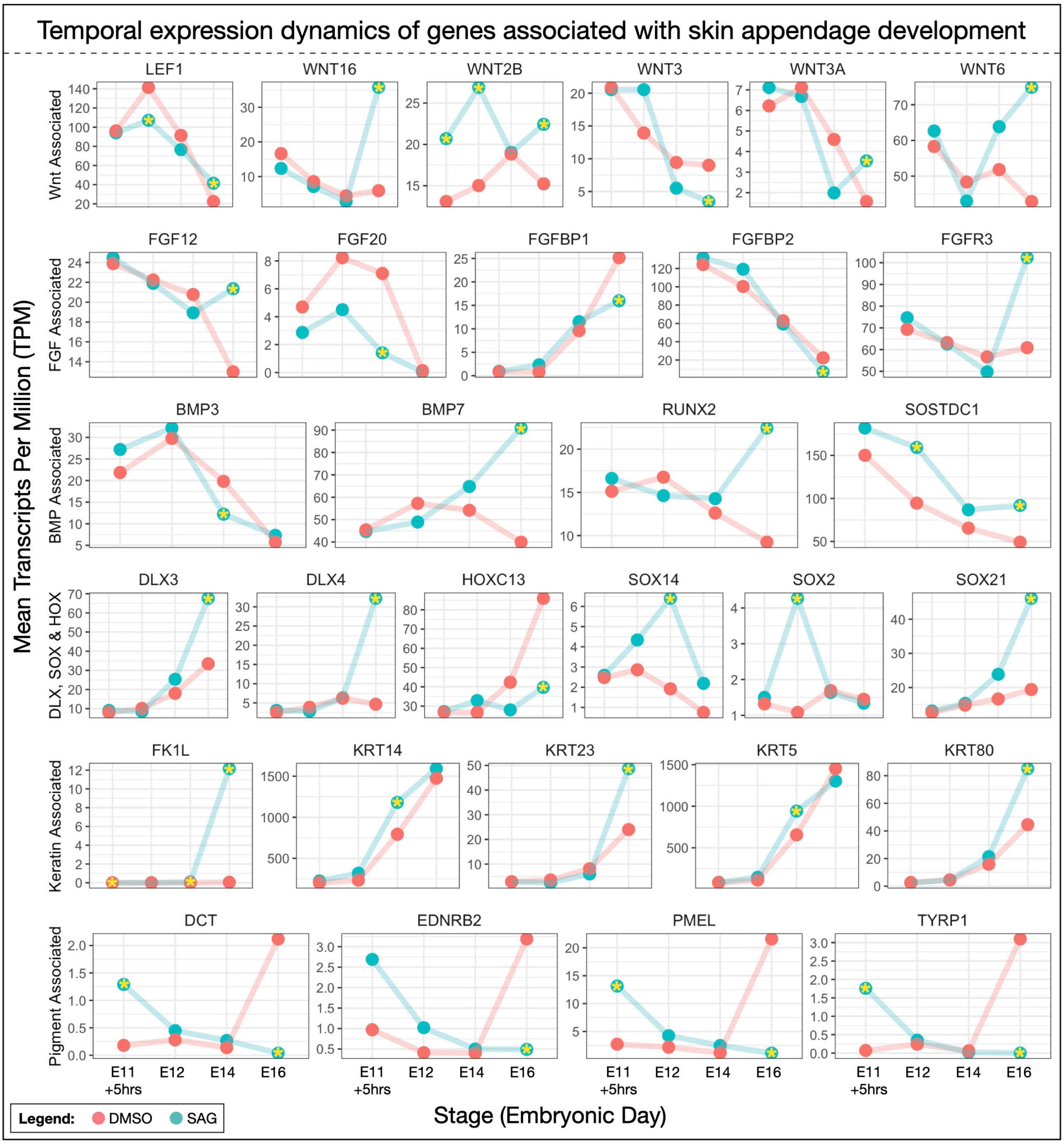

Supplemental figure S6: Temporal expression dynamics of genes related to skin appendage development. Expression values of individual genes are shown at each stage in mean transcripts per million (TPM). Significant differential expression of SAG-treated relative to DMSO control samples is highlighted with an asterisk (FDR adjusted $p \leq 0.05$). Most of the differentially-expressed genes associated with skin appendage development are seen at later stages (E14/E16), whereas Shh pathway members (Fig. 4D) are rapidly and persistently up-regulated after SAG-treatment. Further analyses of these samples are shown in Fig. 4.

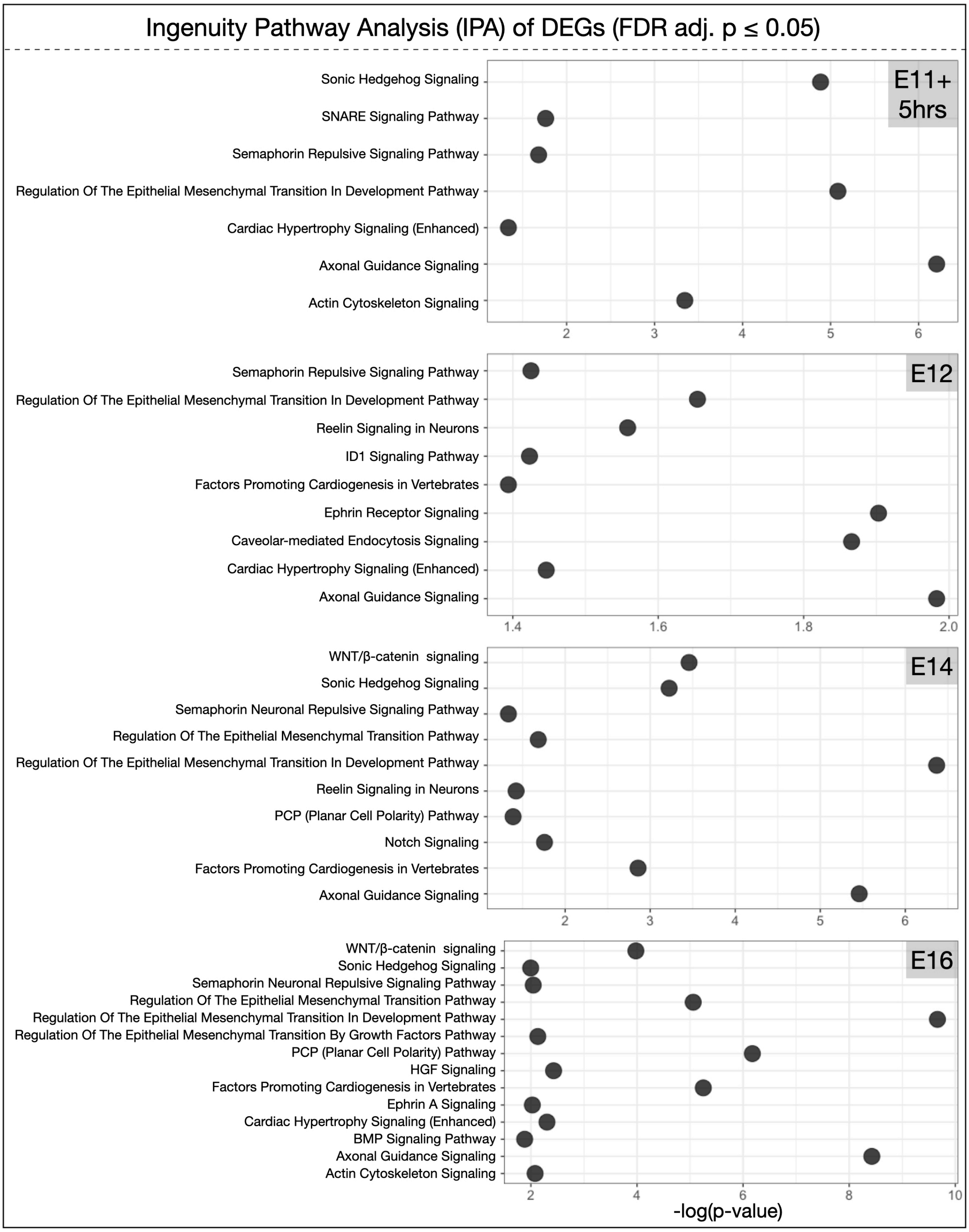

Supplemental figure S7: Ingenuity Pathway Analysis (IPA) of enriched pathways was undertaken for DEGs at each time point. Pathways categorised within the 'Organismal Growth and Development' category are shown, with human and mouse-specific pathways removed. Pathway enrichment significance values are shown using -log(p-value), with a minimum threshold of ≤ 0.05. P-values generated by IPA are calculated by comparing DEGs with manually-curated content of the QIAGEN Knowledge Base, which contains predicted gene and gene pathway interactions. For E11 + 5hrs, E12 and E14, all significant pathways are shown. For E16, only the 14 most significant pathways are shown.

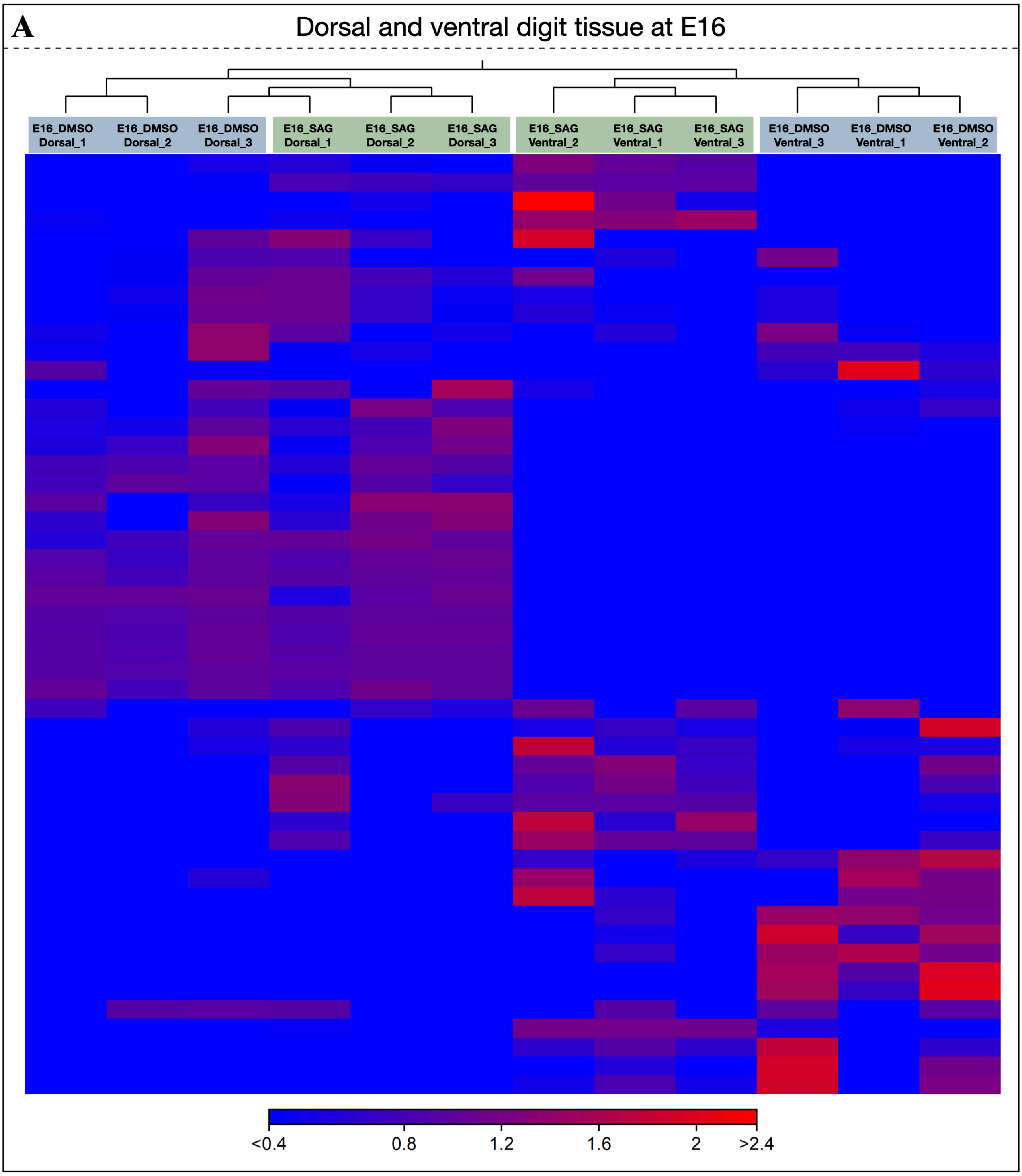

Supplemental figure S8: Heatmap of replicates from RNA-sequencing analyses of SAG-treated and DMSO control digits at E16, dissected into dorsal and ventral tissues. This heatmap is generated with Euclidean distance, clustered by average linkage and filtered by 50 fixed features with a minimum of 10 counts. Both ventral and dorsal tissue replicates cluster distinctly. Furthermore, SAG and DMSO treatment of ventral digit replicates is also distinguished by clustering. Differential gene expression analyses of these samples are shown in Fig. 5.

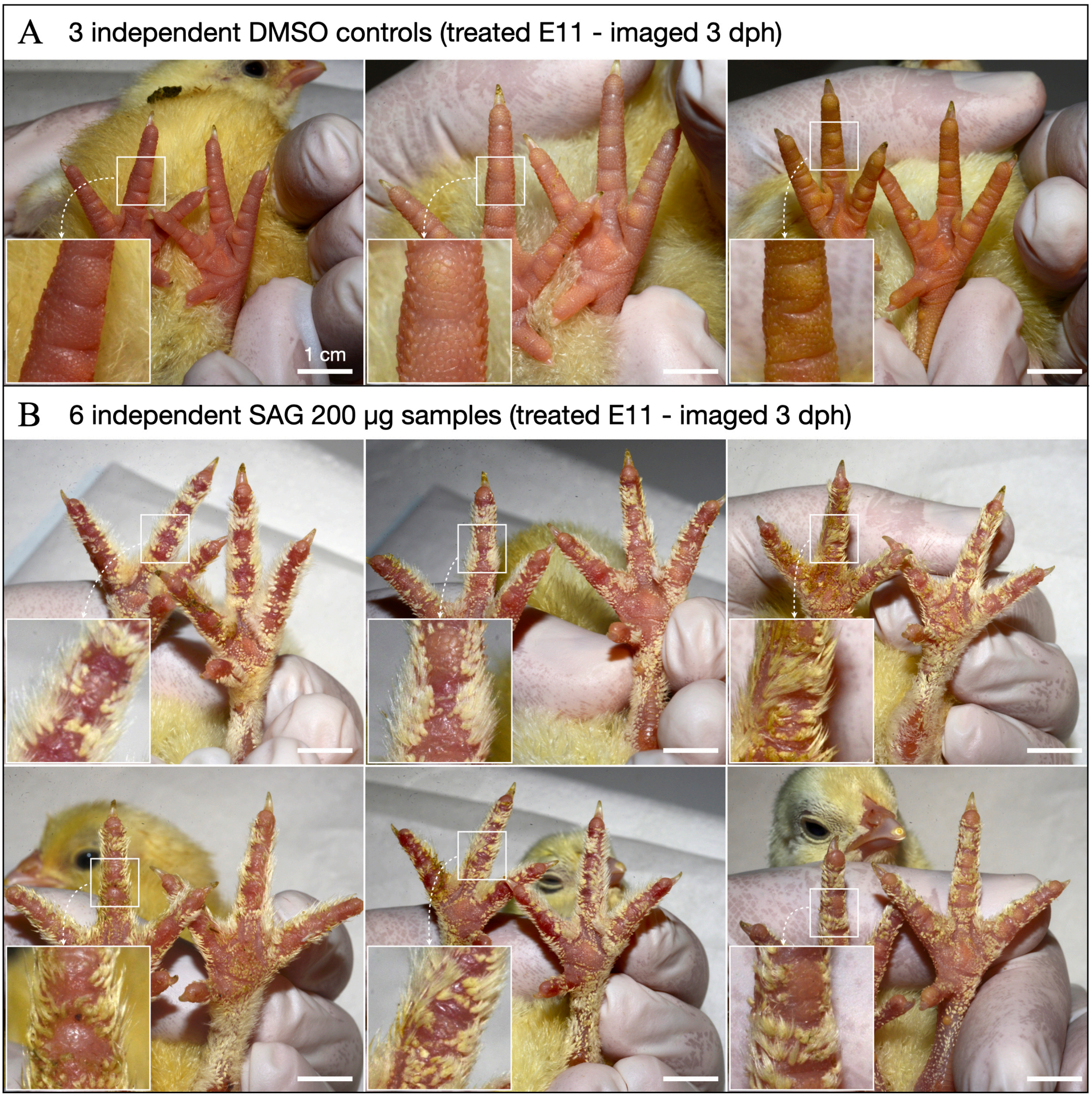

Supplemental figure S9. Replicates of control and SAG treatments (one single injection at E11) and imaged at 3 days post hatching (dph). (**A**) Control samples display normal reticulate scale patterning. (**B**) SAG-treated samples show extensive ectopic feather coverage on the ventral and lateral footpad surfaces. Surviving animals at 8 dph are imaged in supplemental figure S10.

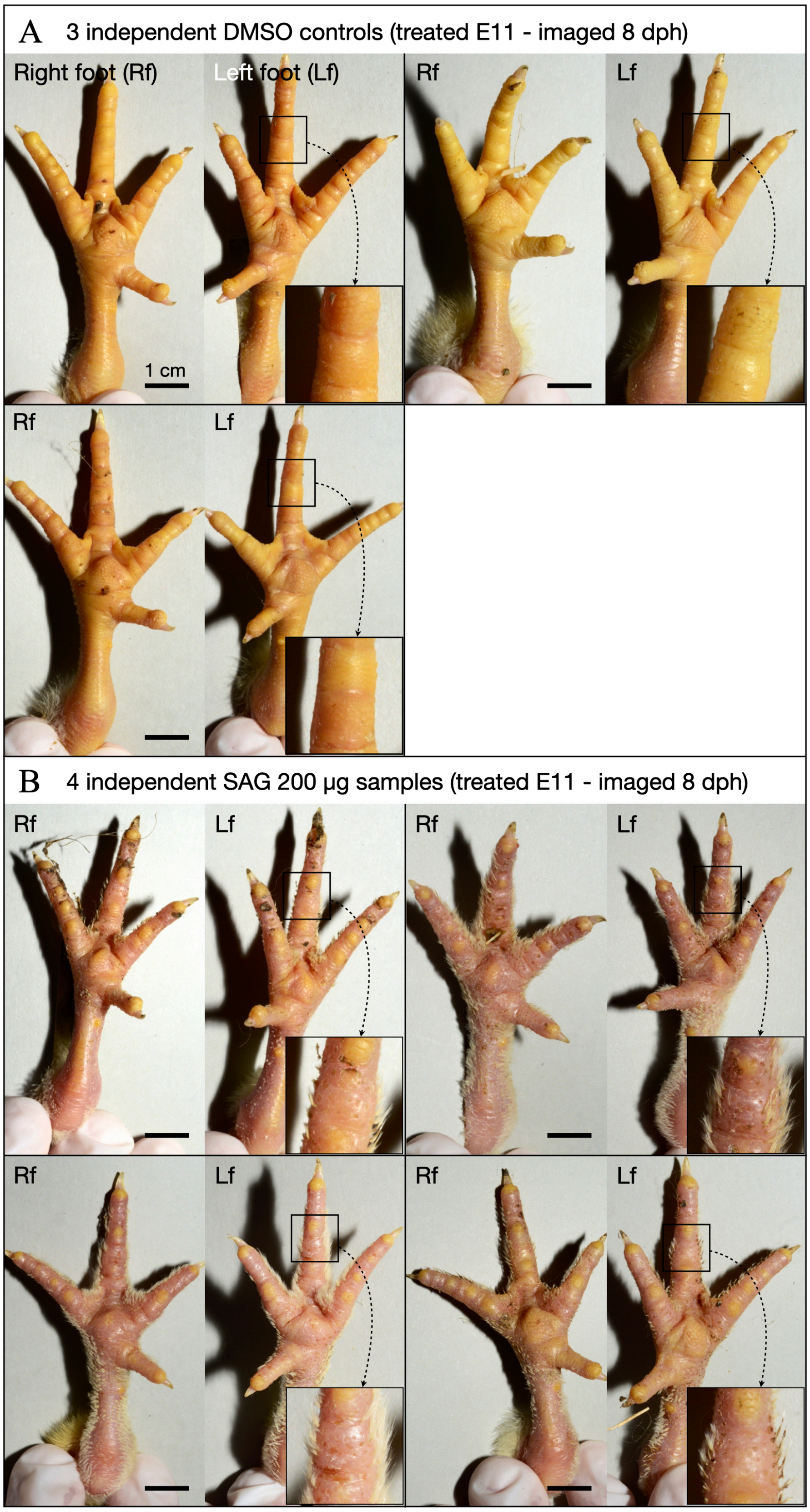

Supplemental figure S10. Replicates of control and SAG treatments (one single injection at E11) and imaged at 8 days post hatching (dph). (**A**) Control samples display normal reticulate scale patterning. (**B**) SAG-treated samples show apparent reduced ectopic feather coverage on the ventral footpad because of abrasion during locomotion. The same animals are shown at 22 dph in supplemental figure S11.

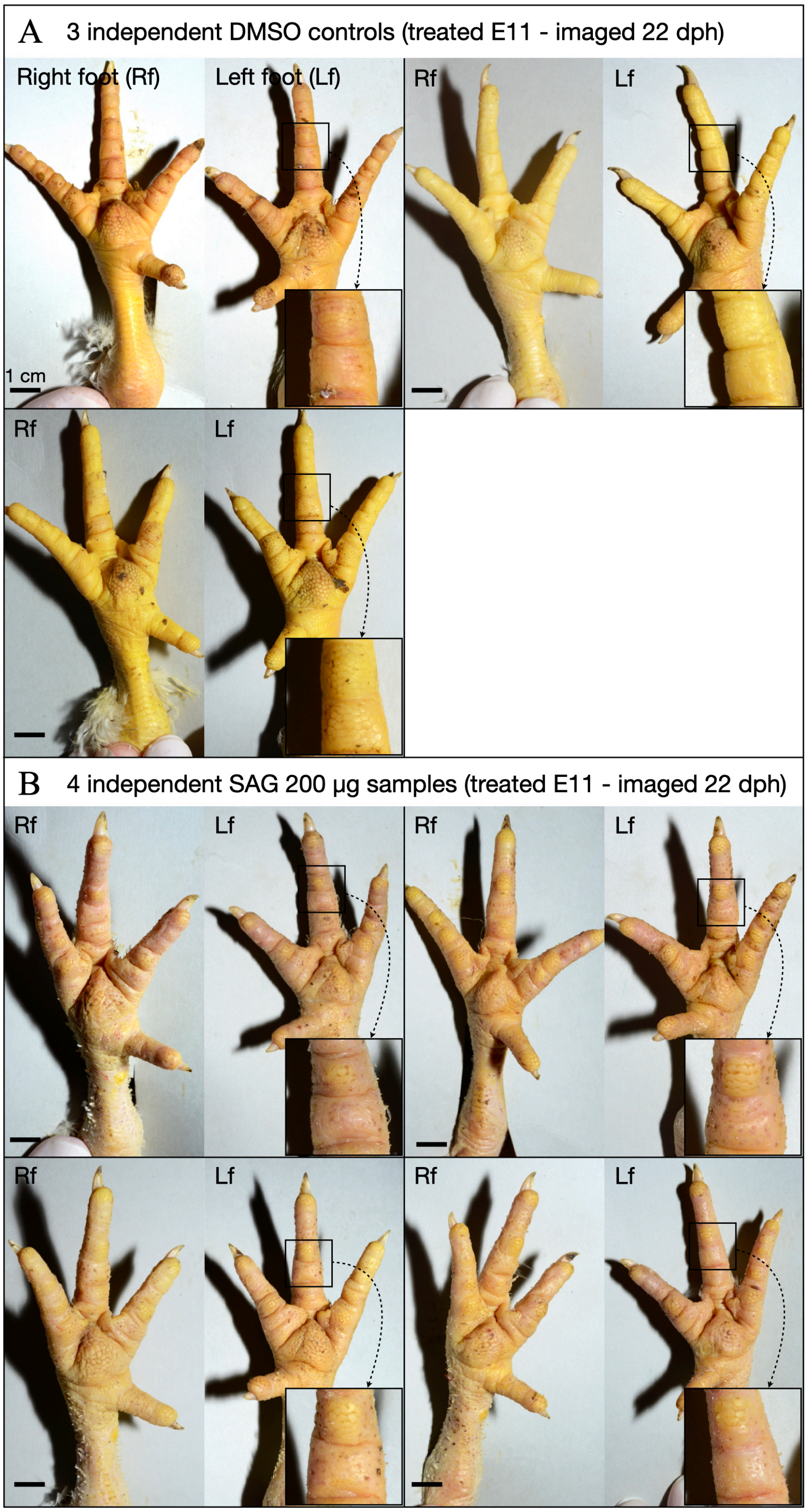

Supplemental figure S11. Replicates of control and SAG treatments (one single injection at E11) and imaged at 22 days post hatching (dph). (**A**) Control samples display normal reticulate scale patterning. (**B**) SAG-treated samples show further apparent reduced ectopic feather coverage on the ventral footpad because of abrasion during locomotion. The same animals are shown at 110 dph in supplemental figure S12.

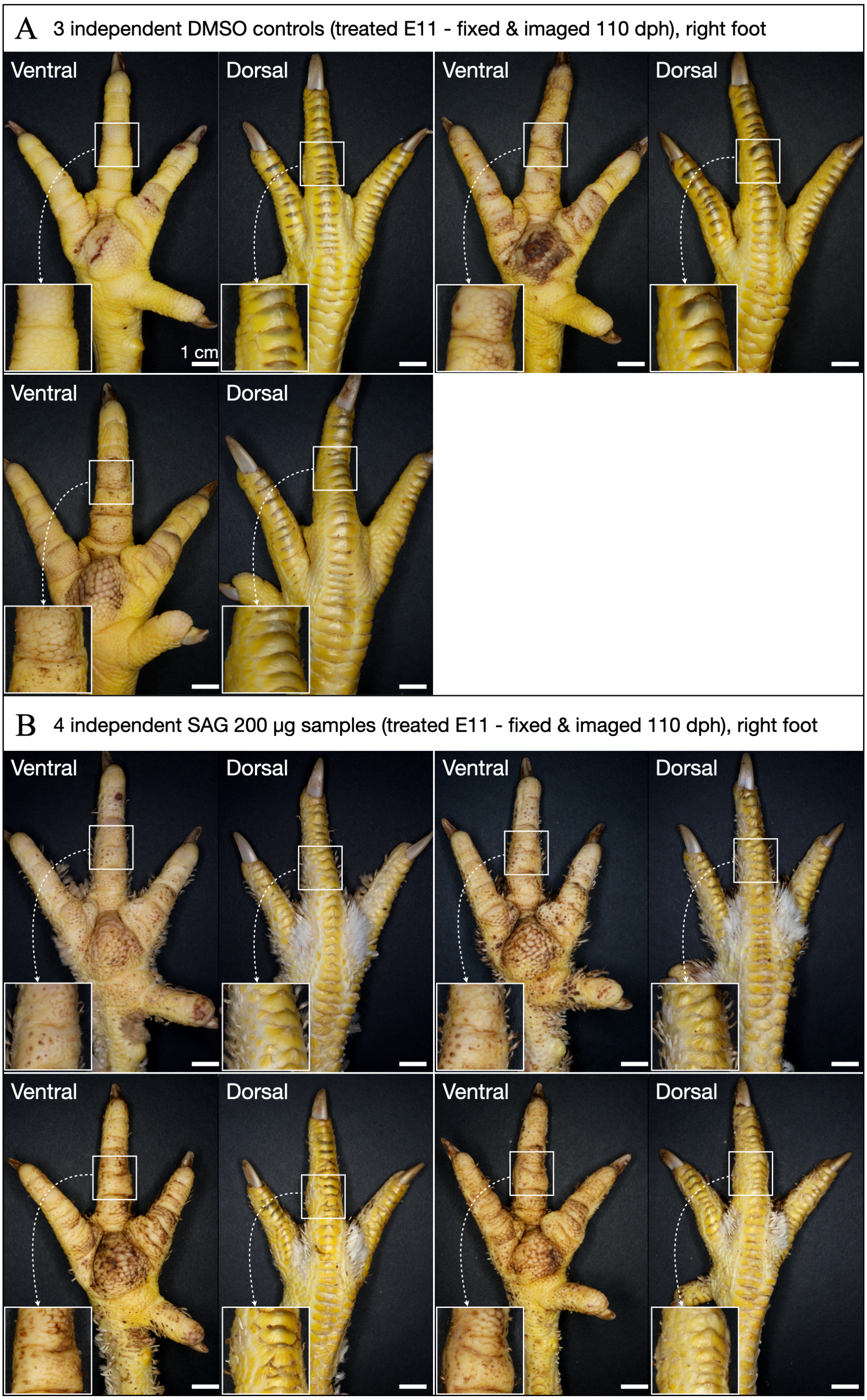

Supplemental figure S12. Replicates of control and SAG treatments (one single injection at E11) followed by fixation and imaging at 110 days post hatching (dph). (**A**) Control samples reveal normal development of scales associated with the foot and metatarsal shank. (**B**) SAG-treated samples exhibit ectopic feathers on the dorsal and lateral foot surfaces and close inspection reveals the presence of feather follicles on the ventral foot surface; the most external part of the associated feathers were likely lost by abrasion during locomotion.

Legends of supplemental movie and supplemental file.

Supplemental movie S1. SAG-induced ectopic feather buds on the avian footpad. Rotating animation showing a 3D reconstruction of LSFM imaging of a nuclear stained (TO-PRO-3 Iodide) SAG-treated embryonic chicken digit sample fixed at E16 (i.e. 5 days post SAG treatment).

Supplemental file S1. RNA sequencing mapped reads. Both expression values for mapped reads and differential gene expression analysis for RNA-seq data presented in Figures 4 and 5 are provided here, in excel table format. All mapping and analysis was undertaken using Qiagen CLC Genomics Workbench.
